# Predicting the current and future distribution of the western black-legged tick, *Ixodes pacificus*, across the Western US using citizen science collections

**DOI:** 10.1371/journal.pone.0244754

**Published:** 2021-01-05

**Authors:** W. Tanner Porter, Zachary A. Barrand, Julie Wachara, Kaila DaVall, Joseph R. Mihaljevic, Talima Pearson, Daniel J. Salkeld, Nathan C. Nieto

**Affiliations:** 1 Department of Biological Sciences, Northern Arizona University, Flagstaff, AZ, United States of America; 2 Translational Genomics Research Institute, Flagstaff, AZ, United States of America; 3 School of Informatics, Computing, and Cyber Systems, Northern Arizona University, Flagstaff, AZ, United States of America; 4 Pathogen and Microbiome Institute, Northern Arizona University, Flagstaff, AZ, United States of America; 5 Department of Biology, Colorado State University, Fort Collins, CO, United States of America; University of Toledo College of Medicine and Life Sciences, UNITED STATES

## Abstract

In the twenty-first century, ticks and tick-borne diseases have expanded their ranges and impact across the US. With this spread, it has become vital to monitor vector and disease distributions, as these shifts have public health implications. Typically, tick-borne disease surveillance (e.g., Lyme disease) is passive and relies on case reports, while disease risk is calculated using active surveillance, where researchers collect ticks from the environment. Case reports provide the basis for estimating the number of cases; however, they provide minimal information on vector population or pathogen dynamics. Active surveillance monitors ticks and sylvatic pathogens at local scales, but it is resource-intensive. As a result, data are often sparse and aggregated across time and space to increase statistical power to model or identify range changes. Engaging public participation in surveillance efforts allows spatially and temporally diverse samples to be collected with minimal effort. These citizen-driven tick collections have the potential to provide a powerful tool for tracking vector and pathogen changes. We used MaxEnt species distribution models to predict the current and future distribution of *Ixodes pacificus* across the Western US through the use of a nationwide citizen science tick collection program. Here, we present niche models produced through citizen science tick collections over two years. Despite obvious limitations with citizen science collections, the models are consistent with previously-predicted species ranges in California that utilized more than thirty years of traditional surveillance data. Additionally, citizen science allows for an expanded understanding of *I*. *pacificus* distribution in Oregon and Washington. With the potential for rapid environmental changes instigated by a burgeoning human population and rapid climate change, the development of tools, concepts, and methodologies that provide rapid, current, and accurate assessment of important ecological qualities will be invaluable for monitoring and predicting disease across time and space.

## Introduction

Tick-borne disease (TBD) diagnoses have steadily risen over the last 20 years across the US, emphasizing the increasing importance of such zoonotic diseases [1]. The rise in TBD has been attributed to several factors, including changes in climate and land-use patterns that influence vector distribution and densities [2, 3]. Historically, TBD surveillance has been conducted at the county, state, or national scale through the National Notifiable Diseases Surveillance System (NNDSS), which requires providers and public health agencies to report cases of specific diseases to the Centers for Disease Control and Prevention (CDC). Several TBDs (Lyme disease, babesiosis, anaplasmosis, tularemia, and rocky mountain spotted fever) are reportable to the CDC [4]. The NNDSS provides important insight into case burdens; however, there are several pitfalls. First, only diagnosed cases are reported, leading to possible under-reporting or over-reporting [5]. Second, cases are reported based on county of residence and not the location of exposure [6]. Third, the presence and population dynamics of the vectors are not considered. These characteristics limit the ability of NNDSS data to predict and explain TBD risk and vector distribution.

Measures of TBD risk, the likelihood of exposure to infected ticks, have traditionally relied on active vector surveillance, requiring researchers to collect ticks from the environment. These collections are most often conducted by either flagging a piece of cloth across vegetation or dragging a piece of cloth across the ground to collect questing ticks [7]. Active vector surveillance has been extensively used to identify and model tick distribution and densities, pathogen prevalence, nymphal infection prevalence, pathogen diversity in sylvatic cycles, seasonality of vectors, risk of spillover, and suitable habitat [8–13]. Active surveillance has long been considered the “gold-standard” for sylvatic TBD surveillance; however, it is resource-intensive, requiring the majority of spatial modeling approaches to aggregate multiple years of data [10, 11], resulting in a loss of temporal resolution (Table 1). For example, some distribution models have utilized active surveillance, collected over three decades, or historical data, collected over the last century, to model current tick distributions. In a stable environment, these methods would likely not impact the outcomes; however, in today’s dynamic world of changing weather patterns, climate, and land use, aggregation of data across multiple years or decades could impact the validity and accuracy of model predictions [14]. Ideally, vector distribution and movement data should be related as closely as possible to appropriate environmental conditions. To achieve that, surveillance methods may be more successful if they trend towards low cost, continuous, rapid, and spatially diverse sample collection.

**Table 1:** Previously published models identifying tick distribution through species distribution models.

Tick Species	Area of Interest	Data Acquisition Method	Years (Range)	Total Size	Modelled	Models	Resolution	Climate Data	Other Data	Citation
*Amblyomma americanum*	Eastern US (770 km from presence points)	Historical Records (Walter Reed Biosystematics Unit)	1995–2015 (20)	14,831 points	181 points	Maxent	~17 km	WorldClim 1.4	None	[15]
*Amblyomma americanum*	Eastern US	Historical Records [16]	1898–2012 (114)	653 Counties	653 Counties	Ensemble	~1 km	WorldClim 1.4	None	[17]
*Amblyomma americanum*	Kansas	Historical Records and Active Surveillance	Not Specified	826 points	682 points	Maxent	10’ or 30’	CliMond	None	[18]
*Amblyomma americanum*	Florida	Active surveillance	2015–2016	33 points	23 points	GLM	100m	WorldClim	Landcover	[19]
*Dermacentor variabilis*	United States	Historical (Smithsonian National Museum of Natural History)	1950–1998 (48)	1,695 points	336 points	Maxent	~1 km	PRISM Climate Group	Land Cover, Elevation	[20]
*Haemaphysalis longicornis*	North America	Historical Records (Walter Reed Biosystematics Unit)	1990–2017 (27)	~11,000 points	304 points	Maxent	~17 km	MERRAClim	None	[21]
*Haemaphysalis longicornis*	North America	Historical	Unknown	~200 points	~200 points	Maxent	10 km	WorldClim	Ecological Zone	[22]
*Ixodes pacificus*	California	Historical Records	1980–2014 (34)	4,546 points	621 points	Ensemble	~1 km	Daymet (1980–2014)	Elevation, Land Cover	[11]
*Ixodes pacificus*	California	Historical Records	1980–2015 (35)	585 points	406 points	Maxent	90 m	California Basin Characterization Model (1980–2010)	Land cover	[23]
*Ixodes pacificus*	Western US (WA, OR, CA, ID, NV, UT, AZ)	Historical Records (Dennis 1998) [24]	1907–2015 (108)	111 Counties	111 Counties	Ensemble	County	WorldClim 1.4	None	[25]
*Ixodes pacificus*	Western US (WA, OR, CA, ID)	Citizen Science Tick Collections	2016–2018	2,332 Ticks	477 points	Maxent	~1 km	Daymet 2009–2018	Elevation, Landcover	Present Study
*Ixodes scapularis*	Midwestern and Eastern US	Historical Records [24, 26]	1907–2015 (108)	1,420 Counties	1,420 Counties	Ensemble	County	WorldClim 1.4	None	[25]
*Ixodes scapularis*	Midwestern and Eastern US	Historical Records [24, 26]	1907–2015 (108)	1,420 Counties	14,200 points	Maxent	~4 km	WorldClim 1.4	None	[27]
*Ixodes scapularis*	Midwestern and Eastern US	Historical Records [24, 26]	1907–2015 (108)	1,420 Counties	1,420 Counties	Ensemble	County	WorldClim 1.4	None	[28]
*Ixodes scapularis*	Minnesota	Field Collections	2005–2014 (10)	122 points	25 points	Maxent	<1km	WorldClim 1.4	Elevation, Landcover	[29]
*Ixodes scapularis*	Ottawa, Canada	Passive Surveillance	2013–2015 (2)	306 points	63 points	Maxent	15 m	None	Elevation, Landcover, Population	[30]
*Ornithodoros hermsi*	Western US	Historical Records	Not Specified	96 points	96 points	Maxent	~1 km	WorldClim 1.4	Elevation	[31]
*Haemaphysalis longicornis*	North America	Historical Records (Walter Reed Biosystematics Unit)	1990–2017 (27)	~11,000 points	304 points	Maxent	~17 km	MERRAClim	None	[21]
*Haemaphysalis longicornis*	North America	Historical	Unknown	~200 points	~200 points	Maxent	10 km	WorldClim	Ecological Zone	[22]
*Dermacentor variabilis*	United States	Historical (Smithsonian National Museum of Natural History)	1950–1998 (48)	1,695 points	336 points	Maxent	~1 km	PRISM Climate Group	Land Cover, Elevation	[20]

Opportunistic citizen science tick collections have the potential to fulfill these characteristics. Citizen science or passive surveillance campaigns have been implemented at both state and national scales by encouraging members of the public to mail ticks to a laboratory for subsequent identification or pathogen testing [32–36]. These programs have been proven to be an efficient method for tick collection and can help to inform on a variety of topics that cannot be easily gathered through a single surveillance technique [32]. Citizen science tick collections have generated large datasets that have provided insights on vector spread, links between tick submission frequencies and reported human TBD cases, seasonality of human-tick exposure, human activity during tick exposure, and pathogen prevalence in submitted ticks [30, 33–35, 37, 38]. Additionally, opportunistic and passive surveillance programs have been used to model or inform on a wide variety of TBD topics. For example, these programs have been used extensively to identify the tick species that people interact with, pathogen prevalence data, and the timing of human-tick exposure at a national level or finer scale [32–34, 36–40]. Recently, these data have also been utilized to accurately model human case data from the number of tick submissions received [35, 38].

A promising avenue of TBD research uses citizen science to collect data on locations of ticks and employs species distribution models (SDMs) to identify the distribution or niche of specific tick species, e.g., this technique created SDMs for *Ixodes scapularis* in Canada and validated predictions with active surveillance [30]. SDMs are commonly used in vector ecology and conservation biology, as they explore variables that may dictate distribution and identify areas potentially supporting current populations. Additionally, SDMs can be projected across future climate scenarios to help predict species distribution changes in response to climate change [41]. These models often incorporate both abiotic (elevation, climate, etc.) and biotic (land cover, other species, etc.) variables as predictors [41]. SDMs have been previously used to model tick distributions of several tick species in North America (Table 1). The majority of these models rely on presence records derived from active surveillance that have been aggregated across time (e.g., over several decades) and/or space (e.g., county-level presence/absence data), inhibiting the ability to identify accurate distributions of changing populations.

Monitoring the current and future distribution of medically important ticks has significant public health implications and can assist in identifying high disease risk areas. On the US West Coast (California, Oregon, and Washington), *Ixodes pacificus*, the western black-legged tick, is the most medically important tick vector and is responsible or implicated in the transmission of *Borrelia burgdorferi* sensu stricto (Lyme disease), *B*. *miyamotoi* (hard-tick relapsing fever), and *Anaplasma phagocytophilum* (anaplasmosis) [42–47]. Here we investigate the application of the dataset from our free national citizen science tick collection program to model the current and future distribution of *I*. *pacificus* across the Western US. We hypothesize that the ability to rapidly collect large datasets with citizen science will provide comparable results to previous SDMs and will supplement traditional surveillance programs.

## Materials and methods

### Citizen science tick collection

Between January 2016 and December 2018, 18,881 ticks were submitted by citizen scientists who contributed to a free tick collection program [32]. The details of the citizen science program can be found in Nieto et al. (2018), but we provide a brief overview here. Citizen scientists were recruited to the program through an initial public relations campaign and a public website (https://www.bayarealyme.org/lyme-disease-prevention/tick-testing/). Additionally, individuals and TBD public awareness groups, not related to the funders or researchers, disseminated information about the program though social media and other advertising platforms. The citizen scientists participated in the program by mailing collected tick(s) and associated data (location of exposure) to the laboratory. The submission form covered information related to locations and characteristics of exposure (date, activity, environment, etc.). Ticks were identified to species using morphological characteristics and tested for the presence of several TBD pathogens.

For the purposes of this study, *I*. *pacificus* submissions from the West Coast with GPS points that corresponded to the associated reported county of exposure were included; though further accuracy of the GPS points was not verified by the researchers. GPS points were converted to correspond to the center of each 1km x 1km grid cell and spatially duplicated grid cells were removed from the dataset, so each 1 km x 1 km pixel had a maximum of a single presence point.

### Predictor variables

We collected a combination of seasonal climatic and land cover variables to build our species distribution model. We had two goals for this analysis. First, to predict the current distribution of *I*. *pacificus* (ecological niche model), we used a model that had both abiotic (climate and elevation) and biotic variables (landcover) to accurately predict the true ecological niche or distribution of *I*. *pacificus* across today’s landscape. Second, we sought to predict *I*. *pacificus’* future distribution in response to climate change. To assess these changes and predict the future distribution we created a climate niche model using the current climate (bioclimatic variables only) and predicted it into the future using CMIP5 climate projections. We were limited by the available variables in CMIP5 climate projections, therefore, we had to remove several variables from the ecological niche model (e.g., land cover, seasonal data). Removal of these variables removes important factors that impact tick distribution, however, including these variables without proper future projections would not allow us to accurately model the potential future distribution.

### Biotic variables (Climate and elevation)

We collected daily measures of three weather variables (minimum temperature, maximum temperature, and precipitation) from Daymet for the years 2009–2018, which were processed to produce nineteen summary statistics for use in the SDMs [48]. All analyses were performed in the statistical package “R,” version 3.5.2 [49]. Daymet files were downloaded and processed in R through the “FedData” package with a native resolution of 1km x 1km [50]. The maximum temperature, minimum temperature, and cumulative precipitation were calculated across each month and averaged across each year. Bioclimatic variables were then derived through the “biovars” function within the “Dismo” package [51]. In addition to these bioclimatic variables, day length, solar radiation, snow water equivalent, and vapor pressure were also incorporated as predictor variables. Similar to the bioclimatic variables, these were downloaded from “Daymet” through the “FedData” package and were averaged across each month and year between 2009–2018. Additionally, these variables were also averaged across each season (Winter: December-February; Spring: March-May; Summer: June-August; Fall: September-November). Finally, an elevation layer was also included with a native resolution of 1km x 1km [52].

### Abiotic variables (Land cover)

Four National Land Cover Database (NLCD) variables were utilized to explain biotic interactions [53]. The native resolution of the NLCD data was 0.03km x 0.03km and was aggregated into four separate rasters with a 1km x 1km resolution, to match the presence data and climate data resolution, by calculating the percentage of each 1km x 1km grid cell that contained (1) forest, (2) scrub, (3) urban low-density, or (4) urban high-density cells. This produced four final variables (% forest, % scrub, % urban low-density, and % urban high density) at a 1km x 1km resolution, while differentiating cells that are heterogeneous. Forest was defined as deciduous forest (NLCD: [41]), evergreen forest (NLCD: [42]), and mixed forest (NLCD: [43]). Scrub was classified using shrub/scrub (NLCD: [52]) and grassland/herbaceous (NLCD: [71]). Urban low density was classified as developed open space (NLCD: [21]) and developed low intensity (NLCD: [22]). Finally, high intensity urban was defined as developed medium intensity (NLCD: [23]) and developed high intensity (NLCD: [24]).

### Species distribution modeling

SDMs were created through “R,” utilizing maximum entropy models accessible through the “ENMeval” package [54]. Maxent models were produced using the maximum entropy algorithm [55, 56] and were assessed through the utilization of area under the curve (AUC) calculations where a value of 1.0 indicates a model that perfectly can classify presence vs. absence (e.g. no false positives or false negatives) [56]. Maxent was utilized as it is specifically designed to handle presence only datasets and utilize background points instead of absence locations. A total of 1600 background points were randomly selected from the study area (California, Nevada, Oregon, Washington, and Idaho). In Maxent models, background points are compared to the presence points to identify specific patterns that are indicative of a species niche. This allows the AUC statistic to differentiate inter-model performance, which is described as ability to identify presence points vs. background points. However, this statistic should be interpreted with caution when assessing the overall model performance (e.g. accuracy), which is heavily influenced by the ratio of presence points to background points and the area of interest [56, 57].

Preliminary models were built to select and identify high contribution variables. Within each stack, the presence and background locations were bootstrapped, from the original datasets, 100 times and subsequently modeled. After locations were bootstrapped, locations were split into training and testing data sets utilizing “checkerboard2” algorithm from the ENMeval package. Feature classes were not restricted in the models, and 0.1, 0.25, 0.5, 1, 2, 4, 8, and 10 were used as regularization multipliers accounting for a total of 48 unique models within each bootstrap iteration. In total, 4,800 models were built for each variable stack (suitable habitat model and climate niche model) during the preliminary analysis.

Variable selection was performed with a similar method to Jueterbock et al. (2016). Differing from Jueterbock et al. our methods incorporated bootstrapped model evaluation and do not serially remove variables, instead our methods utilize moderate to high performing models to remove variables that had low maximum contribution values across all models [58]. Preliminary models with a testing AUC greater than 0.8 were used to remove variables that had a maximum contribution across all models of less than 10 percent. Remaining variables were added to a final stack based on average percent contribution (high to low). Variables with high correlation (< -0.8 and > 0.8 Spearman correlation) to previously included variables were removed (S1 Table).

After predictor variable reduction, presence and background points were again bootstrapped 100 times and 4,800 models were generated for each raster stack (ecological niche model and climate niche model). The model with the highest average AUC across 100 bootstrap iterations was selected as the best model and further analyses were conducted with that model. If any variables had a percent contribution of less than 0.5%, it was removed to simplify the model. The ecological niche model of *I*. *pacificus* was then predicted over the bioclimatic/climate/elevation/landcover variables to identify current suitable habitat for *I*. *pacificus*. Maxent raw output was converted to three sensitivity threshold values by forcing 90%, 95%, or 99% of collected presence points to be predicted as suitable habitat. The 90% and 99% sensitivity thresholds predictions for California were then qualitatively and quantitatively compared to the 90% and 99% ensemble raster from a previous SDM of *I*. *pacificus* in California, generated by active collection of ticks by public health biologists [11]. For the comparison, areas that had 1 or more models predicting suitable habitat in the Eisen et al. ensemble raster were considered as suitable habitat. Our predictions were converted to the same spatial extent (California). We then calculated percent agreement of suitable habitat between the models (i.e. percent area that was predicted as suitable in both models) ($Percent\;Agreement\;of\;Suitable\;Habitat=\frac{Area\;Agreed\;as\;Suitable}{Area\;Agreed\;as\;Suitable+Area\;Pred\;icted\;by\;1\;model\;as\;suitable}\times 100$) and overall percent agreement between both projections (i.e. percent area that was predicted as suitable or unsuitable in both models) ($Overall\;Percent\;Agreement=\frac{Area\;Agreed\;as\;Suitable\;or\;Unsuitable}{Total\;Area}\times 100$).

The climate niche model (bioclimatic variables only) was predicted over future climate predictions using the CMIP5 multi-model ensemble to identify the future climate niche of *I*. *pacificus* [59]. Future projections were computed for two Representative Concentration Pathways (RCP) emission scales (RCP 6.0, and 8.5) at 2.5-degree resolution [60]. Suitable habitat estimates in 2050 were generated through the use of 12 (RCP 6.0), and 17 (RCP 8.5) different climate models. Projections were set to a threshold based on a 95% sensitivity and projections from each RCP value were combined to create a final projection. The final projection shows the number of models that predict suitable habitat across the area of interest. A complete list of models and average bioclimatic variables for the area of interest is available in S2 Table.

## Results

### Citizen science *I*. *pacificus* collection

**Fig 1:**
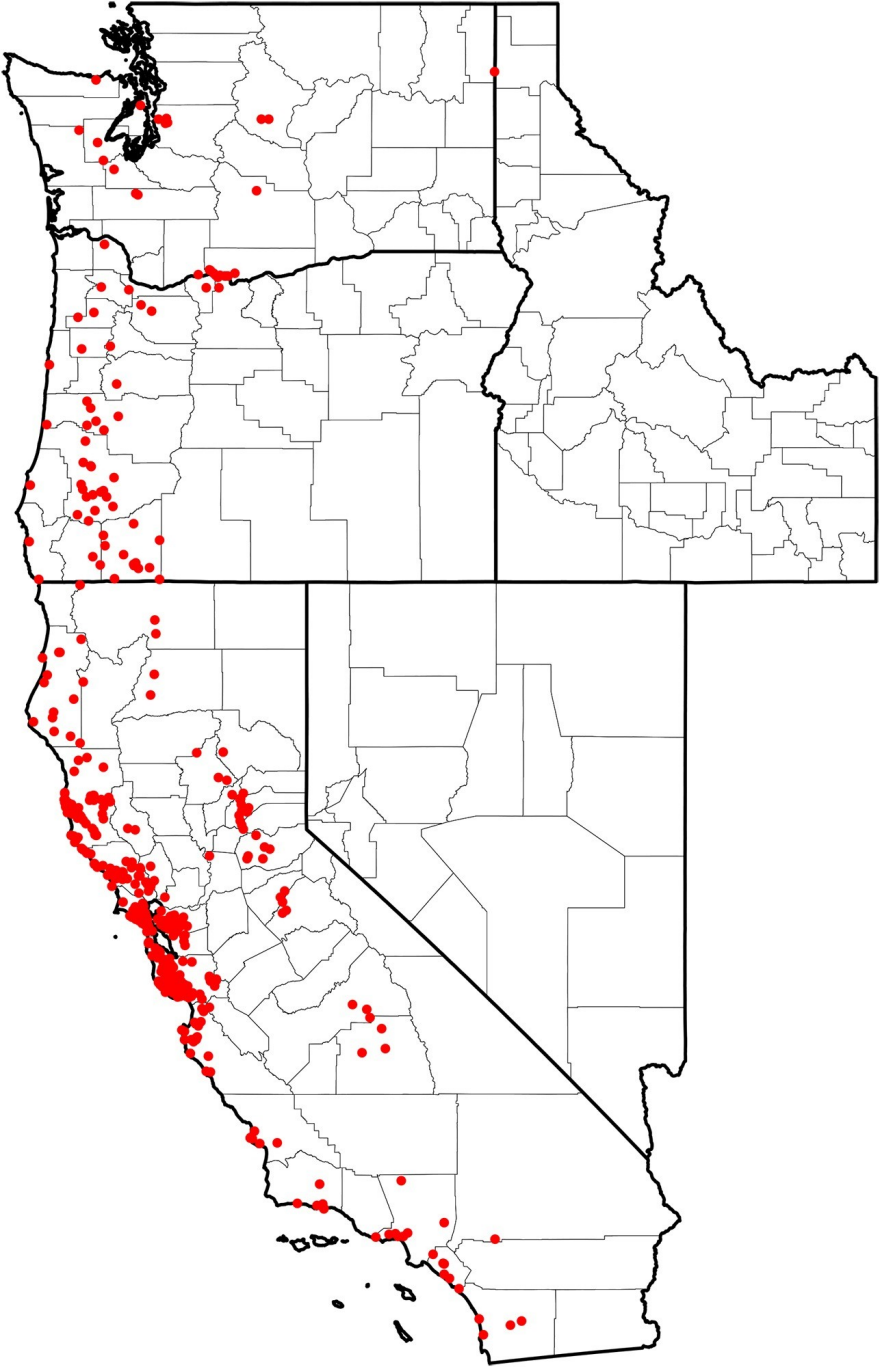
Distribution of collected *I*. *pacificus* (n = 477) corresponding to unique presence points across the Western US.

Of the 2,332 *I*. *pacificus* submitted, 767 (33%) were submitted with valid GPS position information that corresponded to the reported county of exposure. Multiple submissions within each predictor variable pixel (1km x 1km) were removed, producing a total of 477 unique presence points across the study area: California (n = 397), Oregon (n = 54), and Washington (n = 26) (Fig 1).

### Ecological niche model

**Fig 2:**
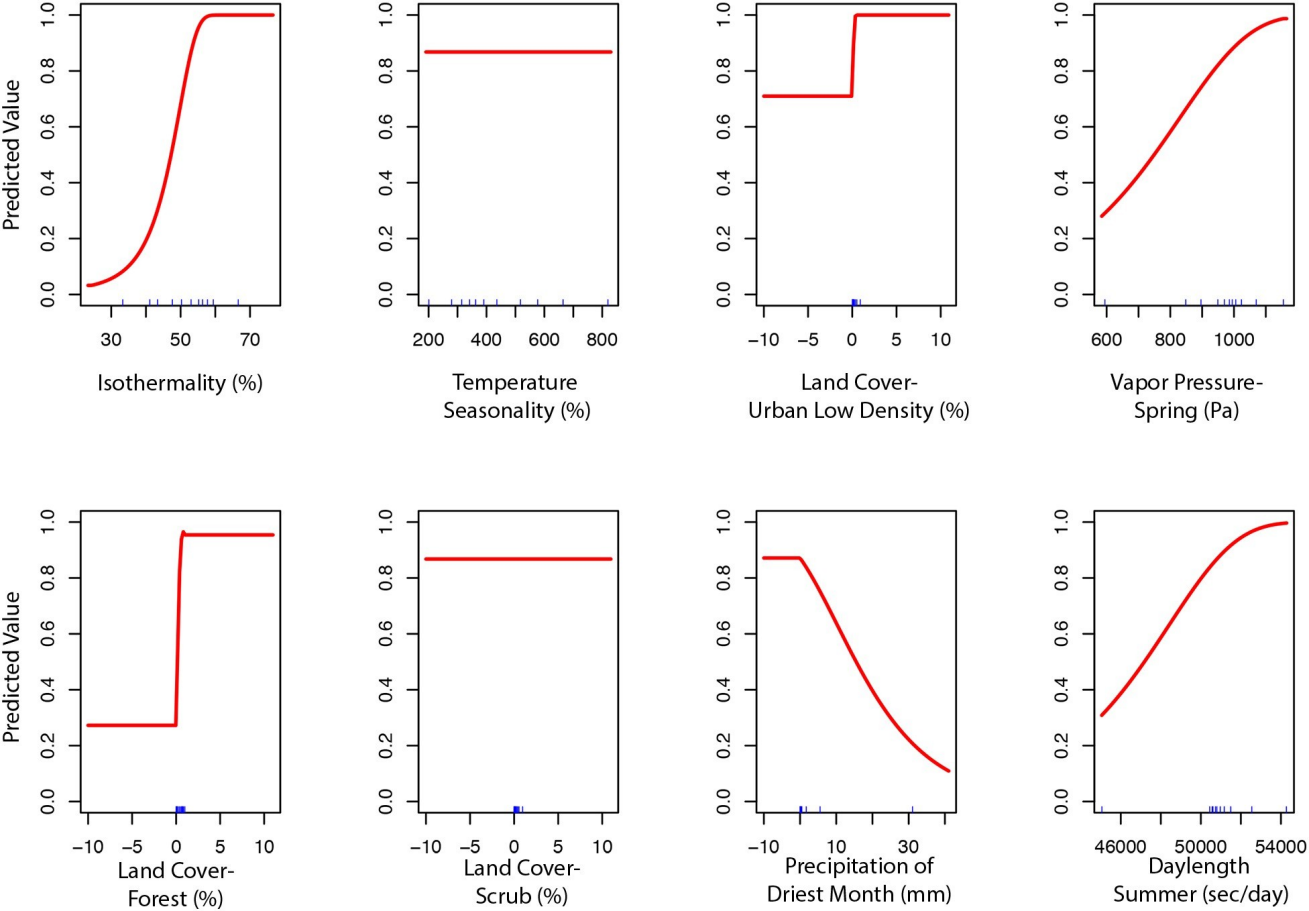
Predicted variable response curves for the suitable habitat model with a linear and quadratic feature class and regularization multiplier of 10.

**Fig 3:**
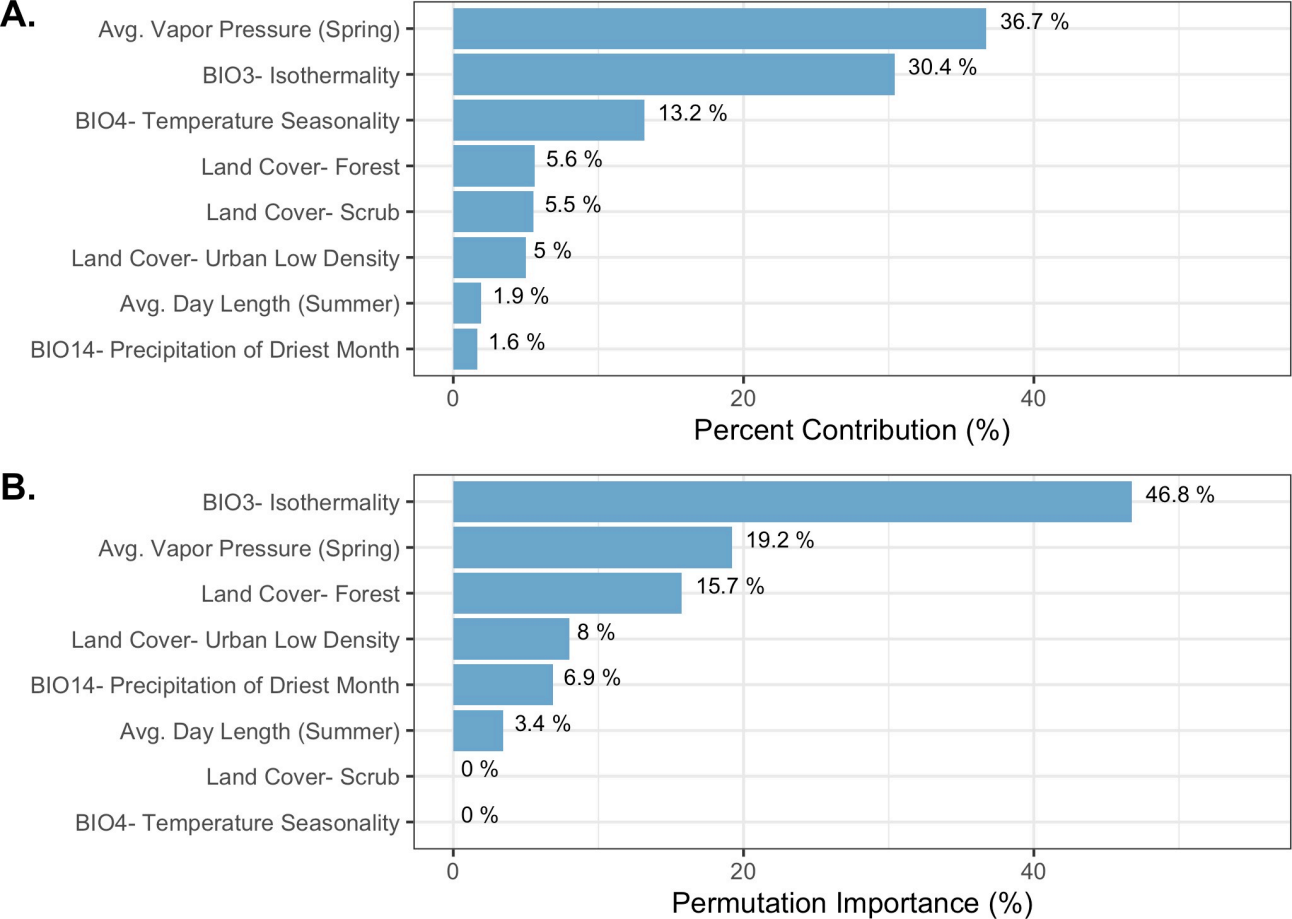
Variable contribution (A) and permutation importance (B) in the final ecological niche model.

**Fig 4:**
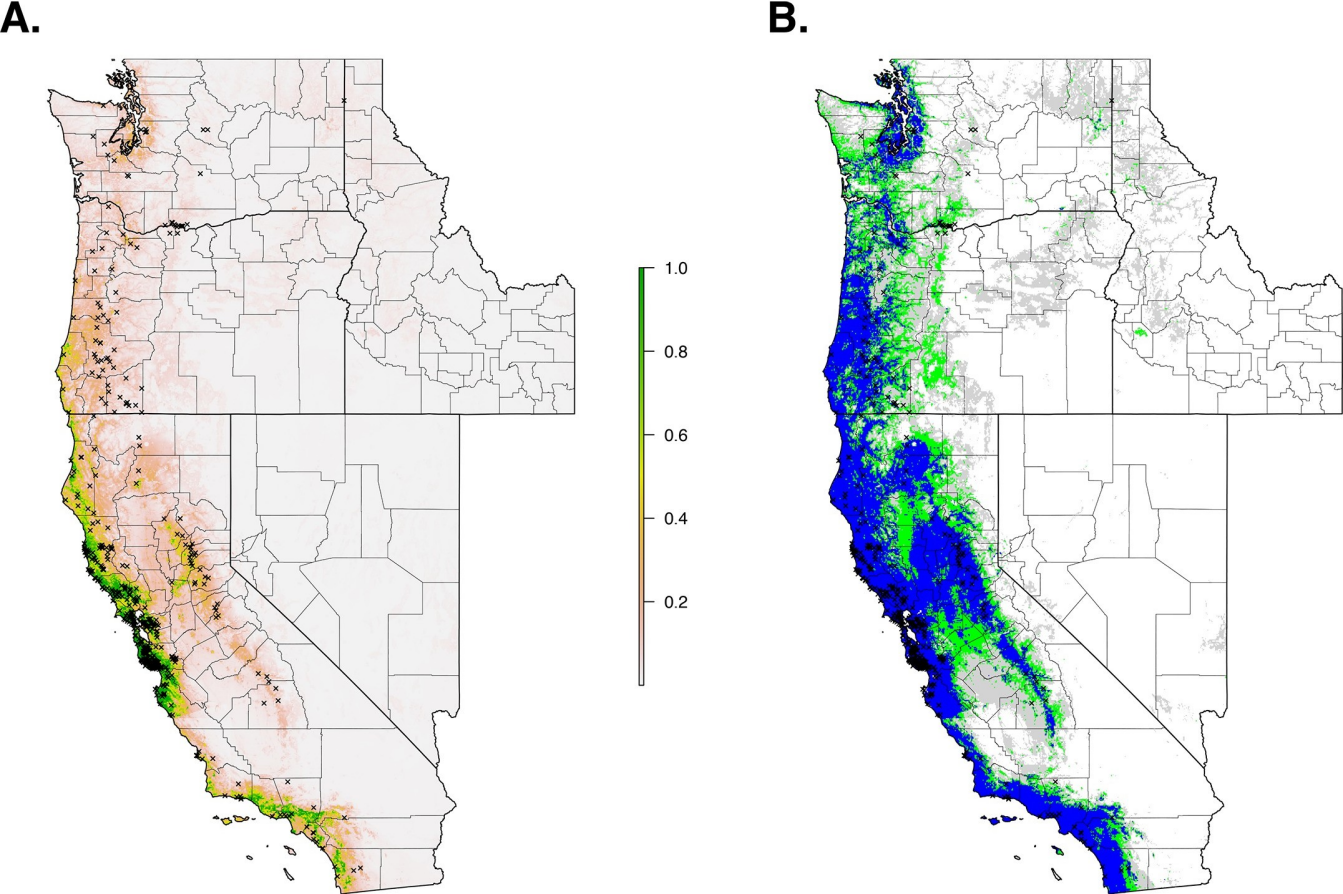
Current suitable habitat of *I*. *pacificus* across the Western US based on climate and land cover variables. Normalized raw output (A) and suitable habitat based on a 90% sensitivity threshold (Blue), 95% sensitivity threshold (Green), and 99% sensitivity (Gray) (B).

The final ecological niche model utilized a total of eight predictor variables: average vapor pressure in the spring, isothermality (BIO3), temperature seasonality (BIO4), land cover- forest, land cover- scrub, land cover- urban low density, average day length (summer), and precipitation of the driest month (BIO14). Average vapor pressure in the spring was the largest contributor (36.7%) to the distribution identifying an increase in the likelihood of suitable habitat with increasing vapor pressure. Isothermality (BIO3) was the second largest contributor (30.4%), predicting increased likelihoods with increased isothermality (Figs 2 and 3; S3 Table). Isothermality (BIO3) had the largest permutation importance with 46.8%, followed by average vapor pressure (spring), and land cover- forest (Fig 3). At a 90% and 95% sensitivity threshold, final predictions showed widespread predicted habitat across the coastal areas of California, western Oregon and western Washington (Fig 4). Additionally, suitable habitat was identified along the Sierra Nevada foothills.

**Fig 5:**
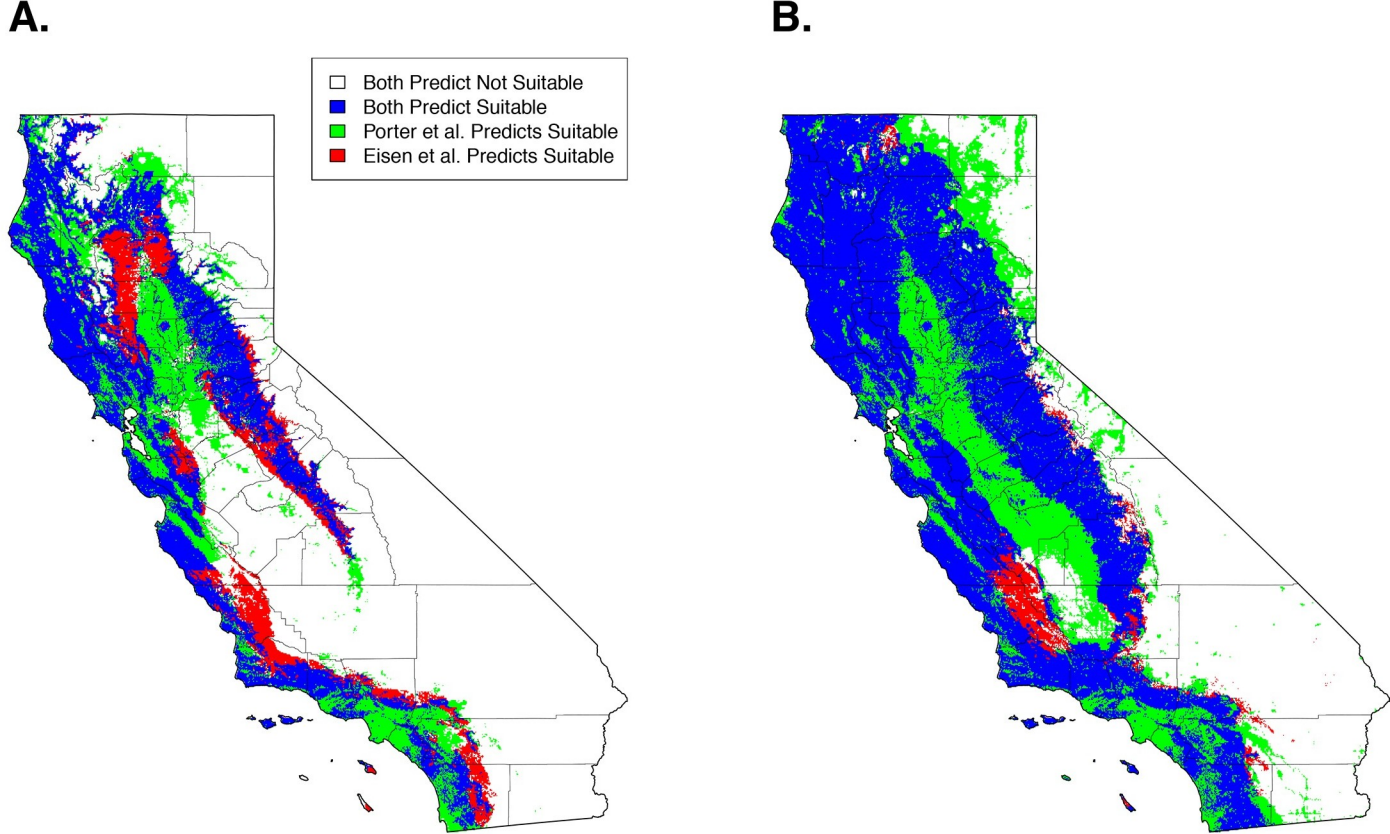
Comparison of predicted suitable habitat presented here (Porter Suitable) and presented in Eisen et al. 2018 at a 90% sensitivity (A) and a 99% sensitivity (B).

Qualitatively, our model predictions of California were similar to an earlier SDM of *I*. *pacificus* distribution that used active surveillance by public health biologists (Eisen et al. 2018) (Fig 5A). Quantitatively, when comparing maps that used the 90% sensitivity threshold (i.e. 90% of the citizen science GPS points are predicted as suitable habitat), overall suitable habitat projections had a 51% overlap of suitable habitat between the two projections. When considering overall agreement, the models had an 80% agreement. The majority of the differences in predictions arise from our model predicting suitable habitat within the Sacramento/San Joaquin Valley and urbanized areas (e.g., San Francisco, Los Angeles) (Fig 5A, green areas). Additionally, Eisen et al.’s predictions identify additional suitable habitat in adjacent areas to areas that were predicted as suitable habitat in both models (Fig 5A, red areas).

Qualitatively, at a 99% sensitivity, the predictions produced here begin to diverge from Eisen et al. predicting widespread suitable habitat across the study area. Quantitatively, suitable habitat predictions had a 64% overlap between both projections and an overall agreement of 76% (Fig 5B). These discrepancies arise from a lack of specificity at high sensitivities that are encountered with the citizen science generated model (Fig 5B, green areas).

To build the final model, the preliminary bootstrap analysis and variable selection identified a total of 16 (16/40) variables that were predictive of *I*. *pacificus* presence. In decreasing importance, these variables were: isothermality (BIO3), temperature seasonality (BIO4), percent of land cover with low density urban, vapor pressure during the spring, percent of land cover with forest, percent of land cover with scrub, precipitation of driest month (BIO14), average snow water equivalent during winter, percent of land cover with high density urban, precipitation of coldest quarter (BIO19), average day length during the summer, average elevation, average vapor pressure during the summer, precipitation seasonality (BIO15), mean diurnal range (BIO2), and average snow water equivalent in the summer. After variable selection, final bootstrap analysis produced a total of 1,832 (38%) models that had an average testing AUC greater than 0.9 and 3,568 (74%) models had an average testing AUC greater than 0.8. Overall, the models with a linear and quadratic feature classes and a regularization multiplier of 10 had the highest average testing AUC (AUC = 0.95, sd = 0.007). After final model selection, several variables still had minimal percent contribution (< 0.05%) and were removed in the final variable selection step; these included: elevation, percent of land cover with high density urban, precipitation seasonality (BIO15), precipitation of coldest quarter (BIO19), mean diurnal range (BIO2), average snow water equivalent in the summer, average snow water equivalent in the winter, and average vapor pressure in the summer. This created the final model with a total of 8 variables (S3 Table).

### Climate niche model

**Fig 6:**
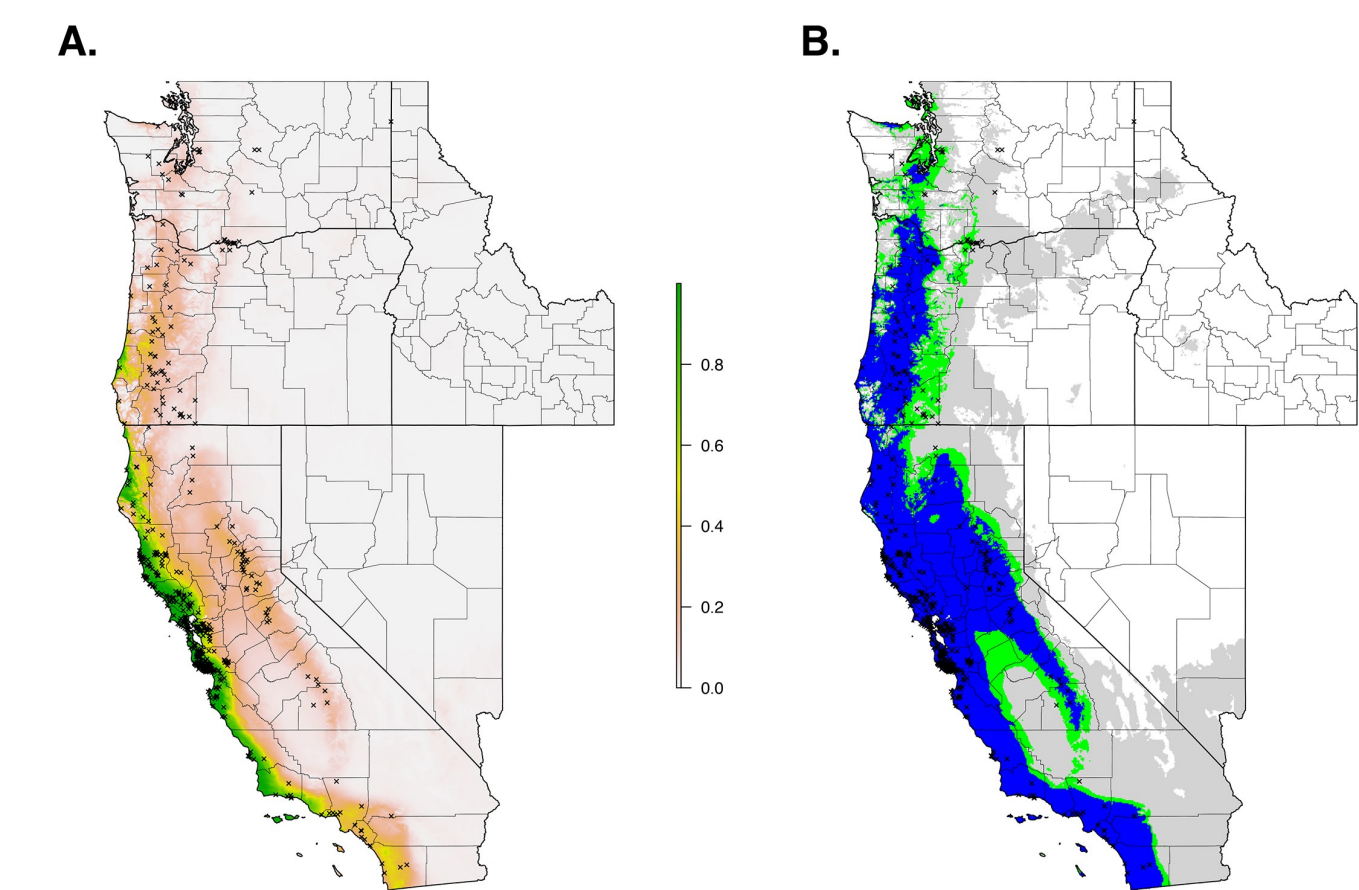
Current climate niche of *I*. *pacificus* across the Western US based on averaged bioclimatic variables between 2009–2018. Normalized raw output (A) and suitable habitat based on a 90% sensitivity threshold (Blue), 95% sensitivity threshold (Green), and 99% sensitivity (Gray) (B).

**Fig 7:**
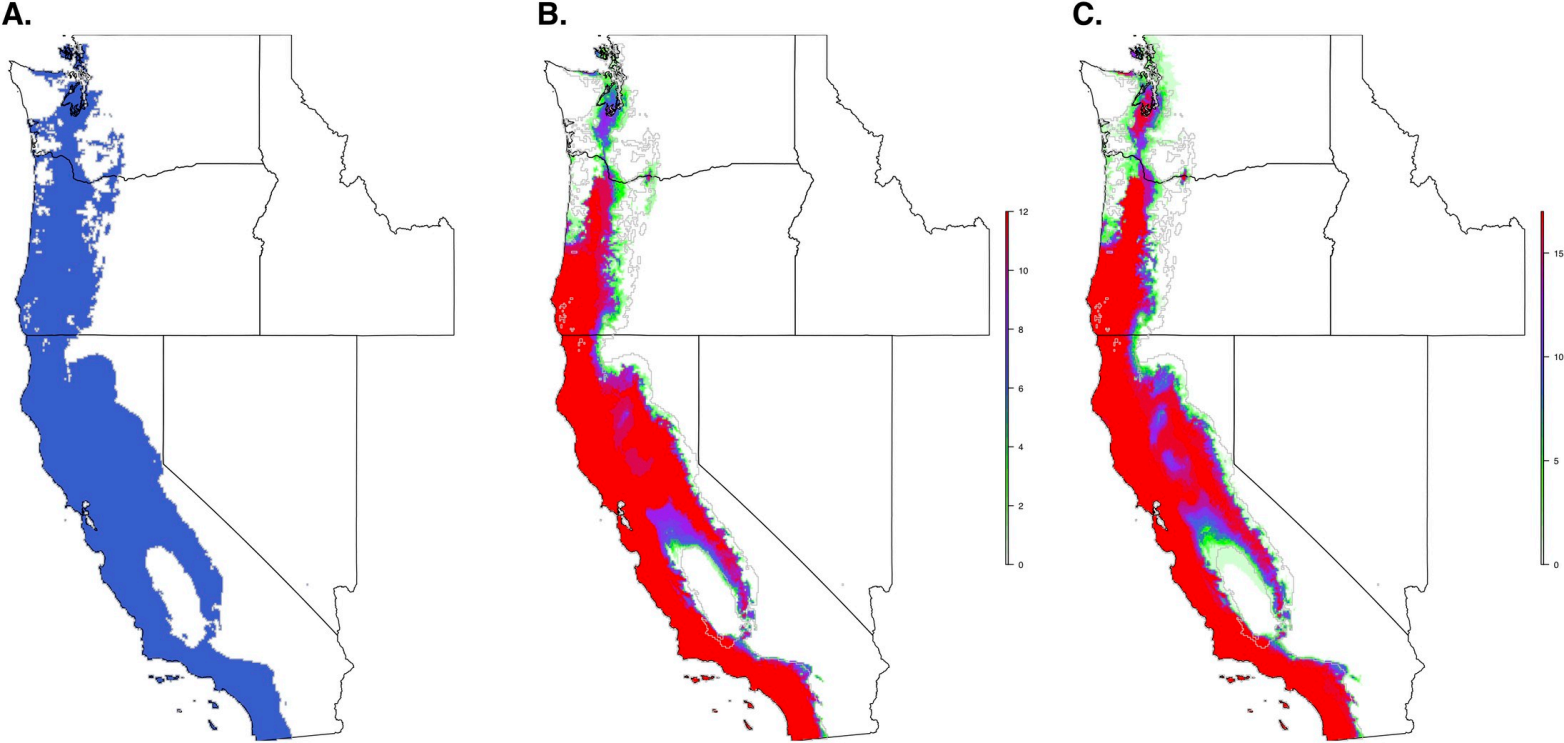
Current climate niche of *I*. *pacificus* based on a 95% sensitivity (A) and number of future climate scenarios (CMIP5) predicting suitable habitat across the *I*. *pacificus* climate niche model in 2050, based on an RCP 6.0 (B) and RCP 8.5 (C) and 95% threshold. The grey line in panels B and C indicates outline of current climate niche.

When we restricted the data set to only the climate variables, so that we could use the model to forecast based on future climate projections, the climate niche model of *I*. *pacificus* showed similar patterns to the ecological niche model; however, in general, the climate niche model identified a more contiguous niche. Additionally, with a 0.99% sensitivity threshold, the model predicted a widespread climate niche across much of the area of interest (Fig 6). Ensemble future predictions using 2050 RCP 6.0 and RCP 8.5 climate projections identified an overall net loss in the climate niche of *I*. *pacificus*. The majority of this loss occurred along the boundaries of the climate niche of *I*. *pacificus* (Fig 7). A small amount of range expansion was predicted in the northern ranges of *I*. *pacificus*, however, this expansion was predicted in a minority of climate scenarios.

**Fig 8:**
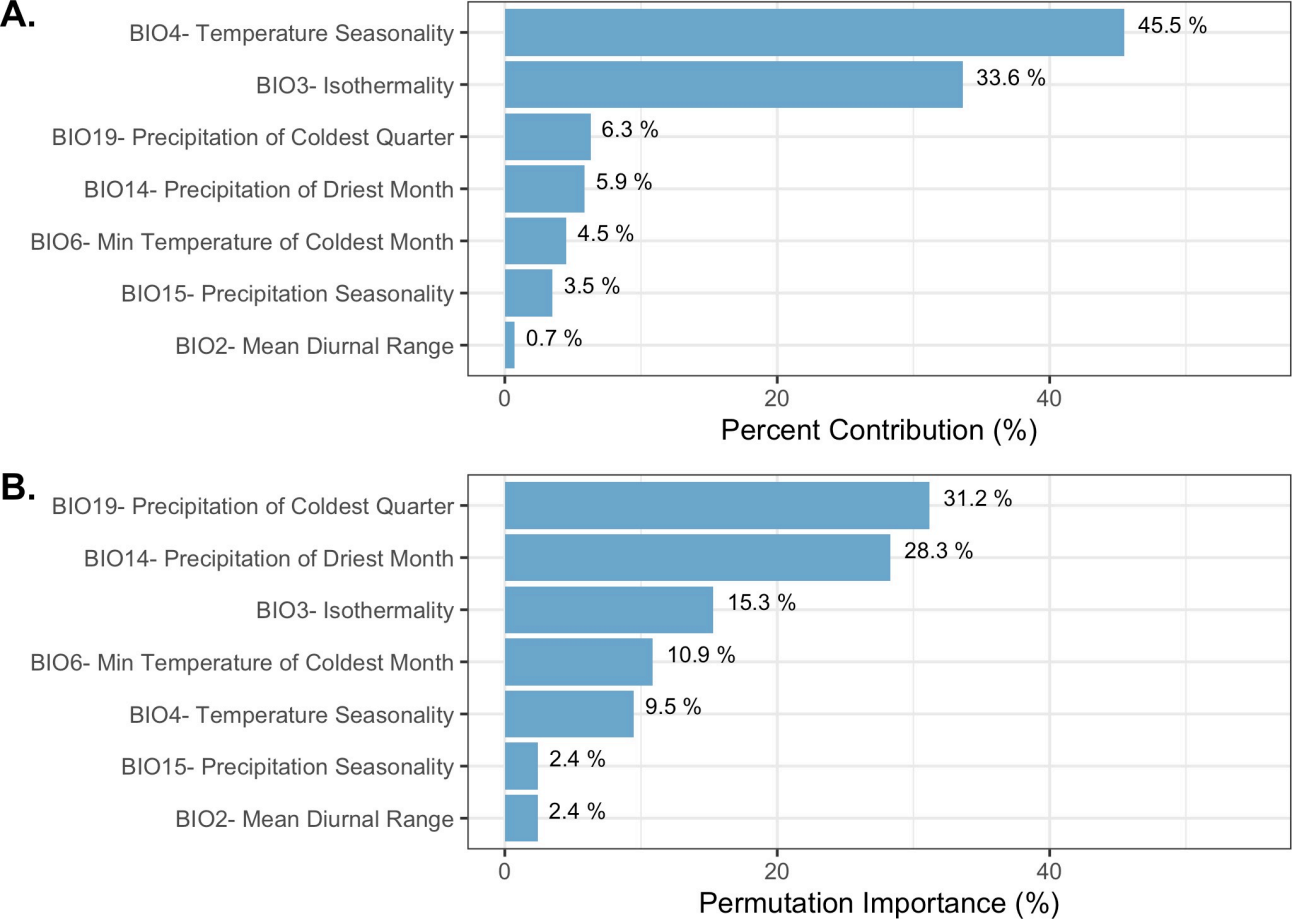
Variable contribution (A) and permutation importance (B) in the climate niche model.

**Fig 9:**
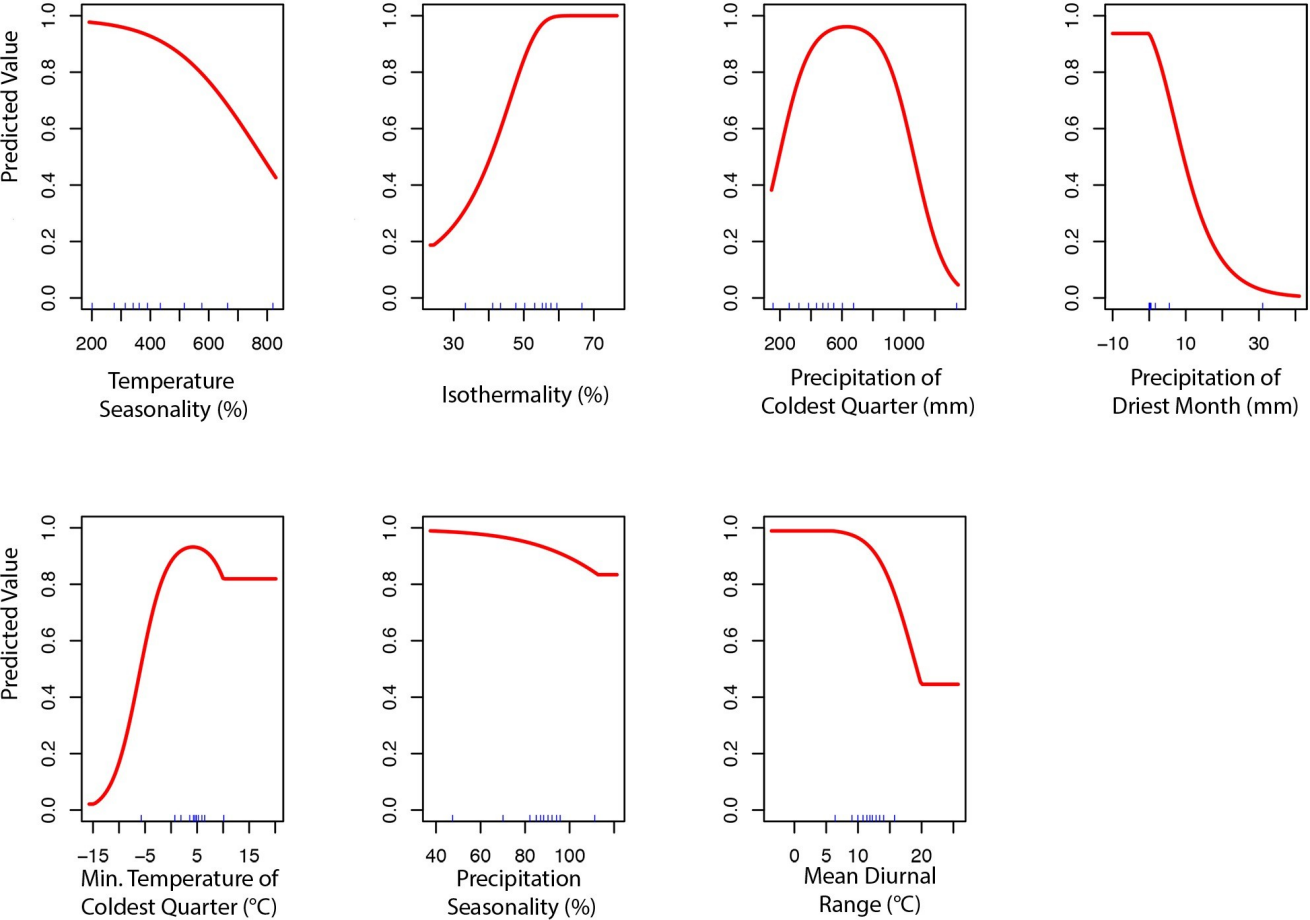
Predicted variable response curves for the climate niche model with a linear and quadratic feature classes and regularization multiplier of 2.

To produce the final climate niche model, the preliminary bootstrap analysis and variable selection identified a total of 7 (7/19) variables that were predictive of *I*. *pacificus* presence and included in the final projection. In decreasing percent contribution, these variables were: temperature seasonality (BIO4), isothermality (BIO3), precipitation of driest month (BIO14), minimum temperature of coldest month (BIO6), precipitation seasonality (BIO15), and mean diurnal range (BIO2) (Fig 8). However, permutation importance included precipitation of the coldest quarter (BIO19) and precipitation if the driest month (BIO14) as the most important variables accounting for ~60% of permutation importance (Fig 8). In general, bioclimatic variables with percent contribution greater than 10% were included in the ecological niche model and the climate niche model. Differences in permutation importance and percent contribution between the two presented models are likely due to additional variables (e.g. land cover and vapor pressure) being included in the ecological niche model. During the preliminary variable selection, the final bootstrap analysis produced a total of 2,033 (42%) models that had an average testing AUC greater than 0.9 and 2,454 (51%) models that had an average testing AUC greater than 0.8. Overall, models with a linear and quadratic feature classes and a regularization multiplier of 2 had the highest average testing AUC (AUC = 0.95, sd = 0.006) and the individual variable contribution was above the 0.5% cutoff for all of the variables (Fig 9; S3 Table).

## Discussion

Traditionally, SDMs identifying suitable habitat for ticks have relied on active surveillance or historical collections that frequently span decades (Table 1). We demonstrate that data from citizen science collections, collected in a two-year timeframe, generated SDMs for *I*. *pacificus* that closely reproduced predictions generated through traditional public health surveillance. Data from previous surveillance methods provide a valuable resource for validating new methods, assessing longitudinal changes, and providing long-term average species predictions. Citizen science collections complement these efforts by providing large and spatially diverse sample collections and can be rapidly generated.

In California, citizen science tick collection based suitable habitat and climate niche estimates, presented here, were similar to predictions based on SDMs that were previously generated through active surveillance over a ~30-year period, identifying suitable habitat and climate niches along the Californian coast, the northern bay area to Sacramento and along the Sierra Nevada mountains [11, 23]. Quantitatively, our predictions were extremely similar at a 90% sensitivity (a threshold that forces 90% of collected presence points to be predicted as suitable habitat). Areas that are not consistent could be artifacts of the modeling processes. For example, Eisen et al. restricted their predictions to areas that were classified by land-cover analysis to be forest, grass, scrub-shrub, or riparian, and excluded agricultural and urban areas. Alternatively, our predictions incorporated the percent of landcover types in a 1km x 1km grid cell as variables into the model, allowing suitable habitat to be predicted in areas that may not have been considered by the Eisen et. al model (Fig 5A). An example of this would be areas that were classified as urban but have enough fragments of natural habitat to support tick populations. Through this modeling difference, we include areas within our predictions that are regarded as unlikely habitats for *I*. *pacificus* (e.g., metropolitan areas and San Joaquin Valley) that may not have enough fragmented habitat to be suitable for *I*. *pacificus* populations. Additionally, Eisen et al. utilized climate data from Daymet aggregated across 35 years, while we used data aggregated across 10 years.

Our 99% sensitivity projections significantly diverged from those presented by Eisen et al., predicting large-scale suitable habitat across California (Fig 5B). This is likely an artifact of extreme sensitivities (> 95%) and citizen science sample collections, which can be prone to inaccurate or mistaken geographical information [32]. With high sensitivities, these samples are forced to be classified as suitable habitat and cause the projections to diverge from previous work. These uncertainties need to be considered when utilizing citizen science collected datasets and can be resolved through using lower sensitivity ranges (< 95%) or through incorporation of targeted active surveillance campaigns to verify tick presence in select locations.

Citizen science approaches have weaknesses: e.g., the impacts of non-structured data collection can produce spatial biases within datasets [61]. These factors are indeed challenging to control for and result in some level of bias. Citizen science tick collections inherently collect ticks that are found by citizens over the collection period; thus, our presence locations reflect where ticks and humans interact, introducing geographical and temporal biases associated with variable sampling effort across space and time, which can over and underestimate distributions. Additionally, our presence points are likely influenced by advertising strategies of the collection program, probably selecting for a high response rate from the San Francisco Bay Area and California. These biases were accounted for within our analysis through the use of random background points and utilizing 90% and 95% sensitivity thresholds, which were more representative of species distributions than continuous predictions. Such spatial biases within presence-only datasets are not specific to citizen science collection methodologies. For example, active surveillance tick collections can suffer from similar biases where ticks are only collected from a few ecological environments (e.g., dense woodland, Eisen et al. 2006), or areas that have established abundant tick populations (for pathogen surveillance); thus, failing to represent tick populations inhabiting different ecological environments (e.g., coastal chaparral), or low-density populations.

Another limitation, specific to citizen science, is that people often do not know precisely where they contacted the tick. Citizen scientists were asked to report where they were exposed to the tick; however, we expect some level of spatial uncertainty. We attempted to account for such uncertainty by using a 1km x 1km spatial resolution and relying on lower sensitivity thresholds. We believe that this variability could drastically impact a fine-scale area of interest modeling campaign (sub-county level) due to the uncertainty of the exact location of the tick exposure, however, at a large-scale (state or regional level), this effect would be relatively minor, and the sample size of citizen science collections likely overpowers this uncertainty spatial uncertainty.

Despite these challenges, citizen science provides a method that supplements other surveillance techniques, providing large-scale sample collection and species distribution monitoring at a spatial and temporal resolution that would be impractical with any other surveillance method. For example, our tick collection program collected specimens from geographical areas (i.e., Oregon and Washington) with sparse active surveillance data. As a result, we could predict an ecological niche of *I*. *pacificus* in Oregon and Washington, an area where suitable habitat for *I*. *pacificus*, via species distribution model, has not previously been defined. Our predicted current ecological niche in Oregon includes a distribution that extends north to south along the western third of the state, consistent with previous active surveillance campaigns which collected *I*. *pacificus* in the western third of the state [62]. Similarly, in Washington, our model predicts patchy suitable habitat along the western third of the state, which is consistent with active surveillance [63].

As tick distributions continue to change due to ecological changes and climate change, predicting the potential future distribution will provide insight into disease/pathogen shifts that could occur, allowing public health agencies to anticipate and combat these shifts. Utilizing future climate predictions (2050, RCP8.5), we identified a 30% loss in the *I*. *pacificus* climate niche. However, the accuracy of future model projections relies on shifts in climate and biotic environments to the extent that the environment is no longer habitable for a target population. This model relies on temperature seasonality (BIO4), which is predicted to increase in the future resulting in a potential loss of suitable habitat in the future predictions (Figs 6 and 7). The shrinking climate niche presented seems to counter the current range expansion of other North American tick populations [3, 64], some of which have been attributed to climate change, bird migrations [65–67], and human actions that facilitate tick movement (i.e., livestock transportation) [21].

## Conclusions

As we continue to grapple with tick-borne diseases, we need to supplement our processes to match the changing and growing needs. Citizen science tick collections and other collection methods, such as mobile smartphone application-based citizen science programs (e.g., The Tick App [68]) and photographic identification of passively collected ticks (e.g., TickSpotters [69]), have the potential to fulfill this need and allow for a variety of questions to be answered that relate to human exposure, clinical case data, and species distributions. Changing ecological conditions will result in an inevitable shift in tick and disease patterns. Tick surveillance is critical for predicting disease risk and must, in order to be most effective, capture small spatial and temporal variations. Citizen science tick collections have the potential to fulfill this need and allow for a variety of questions to be answered that relate to human exposure, clinical case data, and species distributions. Citizen science collections do have some challenges (i.e., spatial biases, biased sampling) that add complexities into analyses; however, the ability to collect extensive and spatially/temporally diverse data, with limited effort, has significant potential across scientific fields.

## Supporting information

S1 Table Correlation matrix of all considered variables. (CSV)

S2 Table Table of current and future bioclimatic variables from Daymet (2009–2018) and CMIP5 database (Future Scenarios) that have been averaged across the area of interest. (CSV)

S3 Table Predictor variables considered and included in the species distribution model of *I*. *pacificus* in the Western US. (CSV)
